# Evaluation of a novel vital sign device to reduce maternal mortality and morbidity in low-resource settings: a mixed method feasibility study for the CRADLE-3 trial

**DOI:** 10.1186/s12884-018-1737-x

**Published:** 2018-04-27

**Authors:** Nicola Vousden, Elodie Lawley, Hannah L. Nathan, Paul T. Seed, Adrian Brown, Tafadzwa Muchengwa, Umesh Charantimath, Mrutyunjaya Bellad, Muchabayiwa Francis Gidiri, Shivaprasad Goudar, Lucy C. Chappell, Jane Sandall, Andrew H. Shennan, on behalf of the CRADLE Trial Collaborative Group, Kate E. Duhig, Natasha L. Hezelgrave, Umesh Charantimath, Chandrappa C. Karadiguddi, Sphoorthi S. Mastiholi, Geetanjali M. Mungarwadi, Feiruz Surur, Lomi Yadeta, Yonas Guchale, Violet Mambo, Sebastian Chinkoyo, Thokozile Musonda, Christine Jere, Bellington Vwalika, Mercy Kopeka, Martina Chima, Josephine Miti, Rebecca Best, Matthew Clarke, Jesse Kamara, Jeneba Conteh, Patricia Sandi, Margaret Sesay, Fatmata Momodou, Julius Wandabwa, James Ditai, Nathan Mackayi Odeke, Annettee Nakimuli, Josaphat Byamugisha, Dorothy Namakula, Noela Kalyowa, Doreen Birungi, Emily Nakirijja, Carwyn Hill, Grace Greene, Adeline Vixama, Paul Toussaint, Grace Makonyola, Doreen Bukani, Monice Kachinjika, Jane Makwakwa

**Affiliations:** 1 Department of Women and Children’s Health, School of Life Course Sciences, Faculty of Life Sciences and Medicine, King’s College London, London, SE1 7EH UK; 2 Maternity Worldwide Community Base, 113 Queens Road, Brighton, BN1 3XG UK; 3 Women’s and Children’s Health Research Unit, KLE Academy of Higher Education and Research Jawaharlal Nehru Medical College, Belgaum, Karnataka 590010 India; 4 Morgenster Mission Hospital, Masvingo, Zimbabwe; 5 Department of Obstetrics and Gynaecology, College of Health Sciences, University of Zimbabwe, Harare, Zimbabwe

**Keywords:** Complex intervention, Maternal mortality, Low resource, Feasibility, Pilot, Implementation

## Abstract

**Background:**

The CRADLE-3 trial is a stepped-wedge randomised controlled trial aiming to reduce maternal mortality and morbidity by implementing a novel vital sign device (CRADLE Vital Sign Alert) and training package into routine maternity care in 10 low-income sites. The MRC Guidance on complex interventions proposes that interventions and implementation strategies be shaped by early phase piloting and development work. We present the findings of a three-month mixed-methodology feasibility study for this trial, describe how this was informed by the MRC guidance and the study design was refined.

**Methods:**

The fidelity, dose, feasibility and acceptability of implementation and training materials were assessed in three representative non-trial sites (Zimbabwe, Ethiopia, India) using multiple-choice questionnaires, evaluation of clinical management (action log), healthcare provider (HCP) semi-structured interviews and focus groups 4–10 weeks after implementation. Simultaneously, the 10 sites included in the main trial (eight countries) collected primary outcome data to inform the power calculation and randomisation allocation and assess the feasibility of data collection.

**Results:**

The package was implemented with high fidelity (85% of HCP trained, *n* = 204). The questionnaires indicated a good understanding of device use with 75% of participants scoring > 75% (*n* = 97; 90% of those distributed). Action logs were inconsistently completed but indicated that the majority of HCP responded appropriately to abnormal results. From 18 HCP interviews and two focus groups it was widely reported that the intervention improved capacity to make clinical decisions, escalate care and make appropriate referrals. Nine of the ten main trial sites achieved ethical approval for pilot data collection. Intensive care was an inconsistent marker of morbidity and stroke an infrequent outcome and therefore they were removed from the main trial composite outcome. Tools and methods of data collection were optimized and event rates used to inform randomisation.

**Conclusions:**

This feasibility study demonstrates that the components of the intervention were acceptable, methods of implementing were successful and the main trial design would be feasible. Qualitative work identified key moderators that informed the main trial process evaluation. Changes to the training package, implementation strategy, study design and processes were identified to refine the implementation in the main trial.

**Trial registration:**

ISRCTN41244132; Registered 24/11/2015.

**Electronic supplementary material:**

The online version of this article (10.1186/s12884-018-1737-x) contains supplementary material, which is available to authorized users.

## Background

Maternal mortality remains a challenge in the post-Millennium Development Goal (MDG) era, especially in low resource settings where in 2013, 800 women died every day from complications of pregnancy and childbirth [1]. The most common causes of maternal mortality worldwide are hypertensive disorders, obstetric haemorrhage, sepsis and abortion complications. There are simple interventions that are readily available to treat these conditions. However, in low-resource settings, delays in presenting to care, reaching care and receiving this care all contribute to high maternal mortality. Measurement of vital signs is the first step in recognising women at risk of deterioration and therefore in initiating life-saving treatments [2]. Despite this, access to accurate equipment to measure vital signs and adequate healthcare provider (HCP) training on escalation pathways are frequently lacking in low and middle income countries (LMIC) [3].

The CRADLE-3 trial aims to determine whether implementation of a novel vital sign alert (VSA) device into routine maternity care at both community and facility levels, will reduce a composite outcome of maternal mortality and morbidity in LMIC. This is a stepped wedge randomised controlled trial (RCT) based in seven low-income and one middle-income country over 20 months. The CRADLE VSA is a semi automated device that measures blood pressure, pulse and calculates the mothers risk of shock. It has been extensively validated for accuracy [4, 5] and usability [6] and the need defined in this context through field work and stakeholder engagement.

The trial development was informed by the Medical Research Council (MRC) guidance for complex interventions [7]. Although the stages of development and evaluation can take many different forms, it is recommended that key uncertainties in the design are systematically studied in a development phase. Procedures should be tested for their acceptability and the likely rates of recruitment estimated to inform sample size calculations. The guidance also states that a mixture of qualitative and quantitative methods is likely to be needed in order to understand barriers to participation and estimated responses. Guidance on how best to undertake this was provided by Moore et al. in 2015 [8].

Whilst the MRC guidance is heavily cited there are few published papers that consider its practical application during this stage especially taking into context the challenges of working in multiple LMIC. Prior to the CRADLE 3 trial start a mixed-methodology feasibility study was undertaken to finalise the intervention and implementation processes which were guided by the Expert Recommendations for Implementing Change (ERIC) project [9]. Potential causal mechanisms and contextual factors that may influence the success of the trial were also identified. A logic model was created to describe these components and to identify the key research questions that inform the process evaluation of the main trial [10]. Through presenting results of this feasibility study, we aim to provide a worked example of the application of the MRC guidance in finalising the subsequent trial protocol and process evaluation.

## Method

The study took place over 3 months from November 2015 to January 2016 with a further 3 months to analyse and adapt the intervention and protocol prior to the trial start in April 2016. It consisted of three key objectives:

1. Exploration of the acceptability and feasibility of the CRADLE programme components and the development of its implementation strategies by a mixed-method evaluation in three non-trial sites representative of the 10 main trial clusters.
2. Collection of primary outcome data in the 10 main trial clusters in order to evaluate the methods of data collection, and assess factors related to the randomisation programme (number of deliveries per month) and the sample size calculation.
3. Utilise results to optimize final CRADLE 3 protocol including training materials and implementation strategy for main trial.

### Setting

Implementation was undertaken in three areas representative of the main trial clusters. These were a convenience sample of facilities meeting the study’s inclusion criteria but geographically distant to avoid contamination of the main trial. All facilities approached agreed to participate. These were sites based around Ramdurg in Karnataka, India, Bishoftu in Ethiopia, and Masvingo in Zimbabwe. In accordance with the 10 main trial clusters (list in Additional file 1) these included one or more secondary or tertiary facilities that provided comprehensive emergency obstetric care and the surrounding primary care facilities that referred to these higher facilities and were urban or semi-urban.

### Participants

The CRADLE VSA was incorporated into routine maternity care. All HCPs working in these services were eligible for training and all women identified as pregnant or within the 6 weeks post-partum period, presenting for antenatal, intrapartum or postpartum care within the three areas were eligible for inclusion.

### Intervention

The intervention, described in accordance with the TiDieR guidance [11], included two key components. The Microlife CRADLE VSA is a novel device that accurately measures blood pressure and pulse [4, 5, 12] and calculates the pregnant mother’s risk of hypovolaemic or septic shock [13, 14]. It has been specifically developed to meet the World Health Organisation’s criteria for use in a low resource setting. A traffic light Early Warning System display alerts users to abnormalities in the vital signs results. The lights are triggered by standard thresholds of hypertension as well as by shock index (heart rate divided by systolic blood pressure) [13], as shown in Table 1. The CRADLE VSA was incorporated into routine maternity care.

**Table 1:** Thresholds that trigger the CRADLE VSA Early Warning System

**Hypertension Thresholds**		
Light and Arrow Results	Category	Blood Pressure (mmHg)
RED LIGHT & UP ARROW	Severe hypertension	≥ 160 and / or ≥ 110
YELLOW LIGHT & UP ARROW	Hypertension	≥ 140 & ≤ 159 and / or ≥ 90 & ≤ 109
GREEN LIGHT	Normal	< 140 and < 90
**Shock Index (SI) Thresholds**		
Light and Arrow Results	Category	Shock Index (HR / sBP)
RED LIGHT & DOWN ARROW	Severe shock	≥ 1.7
YELLOW LIGHT & DOWN ARROW	Shock	≥ 0.9 and < 1.7
GREEN LIGHT	Normal	< 0.9

Primary, secondary and tertiary facilities were allocated devices according to their delivery rate, staffing numbers and number of beds per ward. Pre-existing BP devices were removed from clinical areas, unless existing equipment has functionality designed for that area e.g. repeated automated measures in a high dependency area. This was supported with a CRADLE training package consisting of short animated film, interactive sessions, booklet and posters. There were two sets of training materials available, one for facility HCP and one for community HCP with very limited resources or no formal training.

The CRADLE package content covered:

- How to use the CRADLE VSA.
- Maintenance of the CRADLE VSA.
- Basic overview of clinical assessment and management of pre-eclampsia/eclampsia and shock in relation to the traffic light alerts.

The local implementation team and research team delivered one-off interactive group training sessions lasting 2–4 h to local stakeholders and representative HCP from each of the clinical areas in the cluster. This included a presentation on the background of the CRADLE VSA and the importance of measuring vital signs in pregnancy. This was followed by a demonstration of the features and use of the CRADLE VSA and small group practice using the CRADLE VSA. Training finished with interactive clinical scenarios exploring the use of the CRADLE VSA with available guidance and resources.

Attendees were given training materials and CRADLE VSA devices to disseminate to their clinical areas. The implementation team attended each clinical area over the subsequent days (depending on cluster size) to support dissemination and hold interactive sessions.

The core components of the intervention (provision of the CRADLE VSA devices, animated films, posters and content of the training presentation) were standardized across all sites. The way the core components were delivered was adapted to meet the needs of the site. This intervention can be described as complex [7] because it comprises multiple interacting components that require considerable shift in the behavior of recipients. It is also being implemented at multiple levels of low-resource health services and will affect multiple outcome measures [7]. In the main trial, the intervention will be compared to routine maternity care with clinical assessment and management according to local guidelines. Vital signs measurement is normally undertaken with a variety of blood pressure devices where these are available, which are rarely validated in pregnancy.

### Study design

For this feasibility study, the 10 clusters involved in the main trial collected the primary outcome as defined at that time: a composite of maternal mortality or major morbidity (one of maternal death, Intensive Care Unit admission (or predefined equivalent), eclampsia, stroke, or hysterectomy, with no double counting). Data were collected from existing healthcare facility registers, maternity records, maternal mortality reports and healthcare providers at the discretion of the local research team. Anonymised data were recorded onto a paper form and transferred by the research team onto a purpose-built online database (MedSciNet).

The components of the mixed methodology assessment of the acceptability and feasibility of the CRADLE programme components, undertaken in the three non-trial sites are shown in Table 2.

**Table 2:** Outcomes, method of measurement and time of measurement in the CRADLE-3 feasibility study

Outcome		Method of Measurement	Time of measurement
Feasibility	Fidelity	Duration of training	At training
	Dose	Proportion of staff trained	At training
		Number of facilities included	At training
	Adaptations to fit context	Observation of training	At training
	Understanding of training materials	Questionnaires (*n* = 30 each site)	Post training
		Action Log of Clinical Practice (*n* = 30 each site)	For 1 month post training
Acceptability		Stakeholder Interviews (*n* = 5 each site)	3 months post implementation
		Stakeholder Focus Group (*n* = 1 each site)	3 months post implementation

In order to explore potential mediators of action a purposive sample of HCP were requested to complete action logs of clinical practice to evaluate their referral practice. In these action logs, HCP were requested to record any “yellow” or “red” Early Warning System with the action they took, for example ‘administered anti-hypertensives and referred to hospital today’. This was compared to baseline information of available resources to interpret how HCP had understood and interpreted the training. Questionnaires assessing knowledge of when and how to use the CRADLE VSA and how to interpret the results were filled after training by a random sample of participants.

We undertook semi-structured interviews and focus groups (guides available on request) 4–10 weeks after implementation. This time was selected to allow sufficient experience of the VSA in clinical practice whilst allowing time to make improvements to the intervention within the funding timeline. The aim of these was to further knowledge on the acceptability of the device features gained from a previous observational study of the CRADLE VSA (CRADLE 2) [15] by exploring the feasibility of implementing the CRADLE package and how incorporating it into routine care might change behaviour. Participants were selected through purposive sampling to ensure representation of different HCP cadres in hospital and community care. Participants were approached face-to-face and gave written informed consent to participate in the qualitative study. The interviews were recorded and transcribed verbatim for further analysis and field notes were recorded. Snowball sampling was utilised until data saturation was achieved in each site. Two data coders that were independent to the interviewers undertook analysis using QSR NVivo 11 software (QRS, Vic, Australia). We used the framework method with a coding framework that draws upon the study objectives, logic model and interview guide [16, 17]. New concepts initiated by participants that could not be categorized within the initial scheme were coded using an inductive approach and added to the framework. The COnsolidated criteria for REporting Qualitative research (COREQ) Checklist was used to report the methodology and findings [18].

## Results

### Development of trial tools

We developed the training materials and data collection tools based on the experiences of the previous CRADLE projects [4, 5, 12–14]. It was necessary to create a training package that could be disseminated quickly in multiple contexts and easily understood by every cadre of HCP with different resources available. Therefore, we selected animated film as the main component so that voice overs could be translated at minimal additional cost and it could be disseminated widely onto smart phones by Bluetooth. Feedback from end-users, health service managers and implementation experts was incorporated.

**Fig. 1:**
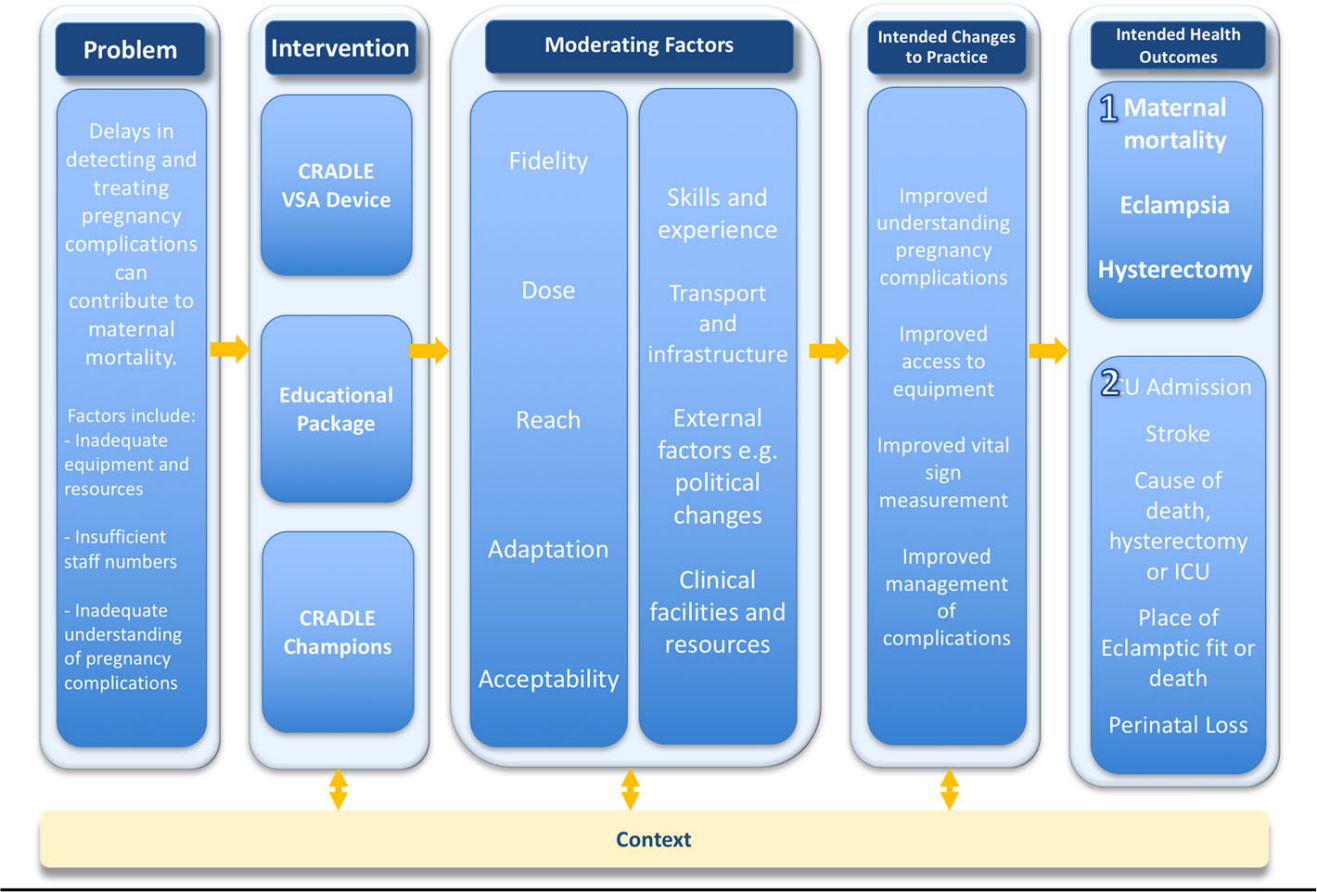
Logic model for the CRADLE-3 intervention

We created a logic model to depict the components of the intervention, how they may interact to produce change and what the anticipated outcome of that change may be (Fig. 1) [10, 19]. This was developed based on the evidence described in the introduction with input of the research team and key stakeholders. These included representative healthcare professionals and service managers from our low-income trial sites and implementation experts. The moderating factors and anticipated changes to practice in the logic model were utilized to inform the process evaluation outcome measures.

### Feasibility of implementation

The intervention was implemented in three regional hospitals, one from each site, together with 61 associated primary healthcare facilities. The proportion of staff trained and the duration of training is shown in Table 3. In India and Ethiopia, we translated the training materials and in India the graphics of the poster and booklets were altered to be more culturally appropriate. In all countries the guidance for management of pre-eclampsia, haemorrhage and shock, which was based on WHO guidelines [20, 21], was adapted to incorporate locally available resources and infrastructure.

**Table 3:** Fidelity and dose of implementation

Study Site	Number of primary health care facilities involved	Proportion of HCP trained	Duration of training	Devices Distributed
Masvingo, Zimbabwe	21	90% (*n* = 92)	8 days	62
Bishoftu, Ethiopia	3	85% (*n* = 37)	3 days	29
Ramadurg, India	37	95% (*n* = 75)	2 days	53

In total, 108 questionnaires were distributed and 97 fully completed by HCP and analysed, 16% by HCP working in primary level facilities and 84% in secondary level facilities. This represents 48% of those trained. Of these, five were doctors, 78 were nurses and midwives and 15 were allied HCP. The majority of respondents had more than 5 years of service (1–5 years *n* = 28, 29%; 6–10 years *n* = 35, 36%; > 10 years *n* = 34, 35%). Results indicated a good understanding use of the VSA and interpretation of results with 73 participants (75%) scoring more than 75% correct answers. HCP working in secondary care scored higher in questions on how to act in response to abnormal vital signs than HCP working in primary care (74% (*n* = 60) scoring over 75% correct compared to 56% (*n* = 9) of clinic participants.

Action logs were distributed to 90 HCP and completed by 68 (76%). The level of detail completed was diverse with many participants failing to complete details on medication given or the time frame taken for action. This was reported to be due to the additional burden of work required to complete these logs and this is explored further in the discussion. Analysis of these records was therefore challenging and results should be interpreted with caution. It was possible to determine that in total, 62 red lights were recorded, indicating severely abnormal vital signs. The majority (*n* = 50, 81%) had optimal care appropriate for the available resources. Of the 12 that did not, five were referred to hospital but within a longer timeframe and seven were reported to look well so no further action was taken.

The action logs demonstrated varying practice in response to a yellow light with a downward pointing arrow. This result indicates a relative low blood pressure compared to the heart rate. In Ramadurg, India, the majority of these patients were referred to higher level care, compared to fewer in Bishoftu, Ethiopia. This variance highlighted ambiguity in the training materials. This was explored widely with stakeholders and changes made to the training materials accordingly. A yellow down light may be caused by multiple clinical causes of varying importance (such as maternal anaemia which can be common in pregnancy) and therefore greater emphasis was made on assessment of the patient’s clinical condition in the final training materials.

### Acceptability of the intervention and implementation strategies

A total of 18 interviews and two focus group were undertaken across the three sites in January and February 2016. Participants were approached face to face and provided written informed consent. The participants had limited prior relationship with the researchers from training and outreach support visits. Individual interviews were undertaken with nine nurses, five midwives, one maternity manager, two health assistants and one doctor. The median age of participants was 25–34 years (*n* = 12) (18–24, *n* = 1; 34–44, *n* = 3; 45–54, *n*-2), the most common number of years of service was 1–5 (*n* = 8) (6–10 years, *n* = 5; 11–15 years, *n* = 2; > 15, *n* = 3). Findings are presented under themes below. Interviews and focus groups were undertaken in private, quiet locations near the participant’s place of work with no non-participant observers to ensure confidentiality and allow for an open discussion. Sessions lasted between 15 and 75 min and were undertaken, translated and transcribed by experienced local research coordinators (clinical background) following training from the trial coordinator and senior social scientist (JS).

### Use of CRADLE VSA in clinical decision making

It was widely reported that the CRADLE VSA package improved capacity to make decisions about which women require treatment and referral. This was true for HCP in the community who initiated referral as well as those working in facilities who initiated treatment or escalated care to senior staff. The suggested reasons for this were varied. HCP who were previously using auscultatory BP devices reported lack of confidence in accurately auscultating the Korotkoff sounds and therefore reported regularly rounding the BP to the nearest ten or delaying taking action. This was alleviated by the digital display of systolic and diastolic blood pressure and pulse on the CRADLE VSA.

*“There, if we get high BP, we used to think and get the BP checked by others, as we had less confidence in that machine. We used to ask others saying that it has shown high BP in our machine and we want to check by you also. Here with this device (CRADLE VSA) we can take decision immediately.” Staff Nurse, Hospital, India*

HCP also reported that the Early Warning System alerted them to abnormalities in the results which may otherwise not be recognised. This was reported to be mainly because in pressured environments with fatigued staff abnormal results may be missed whereas a red light is an instantly recognisable alarm. This, in combination with the training materials was felt to provide valuable guidance in the action required to manage abnormalities. The majority of HCP reported that this has improved the time taken to recognise pregnancy complications and initiate treatment or referral. These views are illustrated in the quote below:

*“Yes, more interventions are done and done in a more prompt manner because of the indicators which one can easily refer to and take action promptly. Interventions are done early or immediately because of the indicators that alert the clinicians. They are forced to act quickly even if they refer, but they will have taken some action” Staff Nurse Masvingo, Zimbabwe*

Whilst the majority of HCP felt that the results are accurate and the lights beneficial, there were two HCP who reported that they did not always act on the lights because they were confused. They felt that the yellow light was reported too frequently when patients appeared well. The quote below demonstrates this:

*“But what we are observing is that for majority of women it is showing yellow with down arrow. But the patient is stable, normal, she is not sick and she is well. In such situations we feel “why it so? The woman is normal, but still why it is showing yellow down”?” Staff nurse, PHC, India.*

Other HCP acknowledged that this was a common result but demonstrated understanding of potential causes and how to manage this result. This finding triangulates the results of the Action Log and is explored further in the discussion.

### Aid of the CRADLE VSA in escalating care and referral

Nursing HCP reported that when they were previously using auscultatory devices they reported doubt or being challenged when referring cases to medical staff. The improved confidence in the vital signs measurement and the understanding conveyed by the training package was beneficial in escalating care to senior staff. This was also true in convincing women and their families of the need for further monitoring or referral.

*“It is helpful in providing immediate care to the patient. As everything is given there (booklets and poster) like the patient has to be referred within 4 hours or within 1 week...etc., we call the attenders and convince them, to refer the patient at the earliest.” Staff Nurse, Gokak, India*

The majority of HCP reported an increase in the number of vital sign abnormalities detected; however, the reported impact on the rate of referrals varied between countries. All participants from India reported an increase in the number of referrals. The number of hypertensive patients referred was increased as mild hypertension was considered more seriously. The number of patients with low blood pressure detected and referred was also noted to increase. In Zimbabwe and Ethiopia responses were mixed with some reporting a reduction in referrals due to improved understanding of vital signs and confidence in initiating treatment peripherally. A quote to illustrate this is:

*“because of the indicators the referrals have decreased, now we know when to refer and we now don’t refer all patients for the sake of referring.” Nurse, Clinic, Zimbabwe*

### Use of results to finalise implementation strategies

The MRC Guidance and supporting documents highlight that the pilot data should be used to shape the intervention and implementation strategies. Following the completion of this feasibility study, the experiences of implementation and input from a stakeholder meeting across all sites in February 2016 were used to agree a number of changes prior to the main trial. The choice of implementation strategy was guided by the Expert Recommendations for Implementing Change project [9] with the aim of improving understanding of the training materials and overcoming identified barriers to use. Table 4 presents the key issues experienced through the feasibility study alongside quotes to support this and the implementation strategies or changes we have selected.

**Table 4:** Barriers to measuring the vital signs and using the VSA and actions made prior to the main trial

Issue from Feasibility study	Supporting quotes from Interviews and Focus Group	Changes required after feasibility study
Understanding of yellow light with down arrow	*“When the pregnant woman comes to us she is well and it shows yellow down (laughs); in that case how to interpret?” Staff Nurse, India*	- Training materials updated to explain the reason for each colour light. - Guidance updated to place greater emphasis on how to assess the woman to decide *if* further action or referral is required.
Problems with charging the VSA	*“… some workers say that the battery life is good but I have to charge it for daily. If I have to go for BP check-up today I have to charge it first.” Staff Nurse, India*	- Interactive training session developed which incorporates guidance on charging and accountability for charging. - Including explanation that more than 100 readings can be taken even when the battery low sign is showing and overcharging will damage the battery.
Provision of Charger	*“the package should include the charger rather than USB cable only so that we can charge it easily” Staff Nurse, Zimbabwe*	Chargers provided in addition to the cable that comes as standard in the package.
High staff turnover / incapacity to train all staff at once	*“Initially, we faced difficulty. There were nurses using it during the night shift and they were not trained.” Staff Nurse, Bishoftu*	Designated CRADLE Champions in each facility identified to provide ongoing local training and support for CRADLE VSA
Unsupportive seniors	*“Recently during an ANC clinic he inquired which device we are using; we said “we are using the CRADLE device given by the research unit” then he said “oh… it shows yellow down to all pregnant women, so better do not use it”.” SN, India*	- Designated CRADLE Champions identified to provide ongoing local training and support for CRADLE VSA - Engage Local Opinion leaders prior to implementation
Need for equipment	*“…no BP machines at all, the one we had was no longer working.” Staff Nurse, Zimbabwe*	Ensure adequate supply of VSA available

A key implementation strategy that we selected as a result of the feasibility findings was the identification and training of CRADLE champions. These are clinical staff and local leaders who receive more in-depth training about device and are equipped with the tools to support the use of the VSA in their area. Whilst we wanted the trial to remain pragmatic and replicable, this strategy was chosen to support ongoing local training and build local capacity for maintenance. The importance of the support of the implementation team on the effectiveness of the intervention was also noted during the study and added to the logic model as a core component of the intervention. In order to maintain the pragmatic trial design, it was decided not to quantify this support but for the research team to observe it during the routine monthly site monitoring and be aware of its potential impact.

### Main trial primary outcomes

As the main trial has a stepped wedge design with an intervention incorporated into routine care, cluster level consent instead of individual was appropriate. This was agreed as preferable by all sites during the development work. All ten trial sites gained ethical and local approval for participation in the main trial. Nine of them achieved this before or during the feasibility phase and were therefore able to commence data collection, with six sites completing a full 3 months of data collection. During this formative phase a total of 844 outcome events (any one of eclampsia, maternal death, hysterectomy, ICU admission or stroke) occurred in 681 women from nine of the ten sites. On average, the number of women experiencing events each month varied from five in Gokak, India to 63 in Zomba and South-Eastern Malawi (median 19.6, standard deviation 19.6). The most common outcome was eclampsia (*n* = 386, 46%) followed by ICU admission (*n* = 271, 32%), hysterectomy and maternal death (*n* = 96, 11%; *n* = 91, 11% respectively) and stroke (*n* = 2, 0.2%). The tools for data collection were found to be appropriate. Methods of data collection were discussed and optimised based on the existing resources available in each site. Outcomes were triangulated across multiple sources (including referral registers, ward registers, patient records, local mortality and morbidity records, active case finding) to ensure data completeness and all outcomes checked to avoid double counting.

These outcomes had been intended as unequivocal markers of severe maternal mortality and morbidity. However, during the process of data collection it became apparent that ICU admission was dependent on availability of services which varied greatly between clusters and therefore this was not a reliable proxy marker of maternal morbidity. The number of strokes reported across all sites was just two, both from one site. It was therefore prospectively decided that for the main trial the composite primary outcome would include only eclampsia, maternal death and emergency hysterectomy. Given that the size of clusters varies greatly, it was agreed that the number of deliveries would be counted as a surrogate denominator so that results for the main trial could be presented as an event rate per 10,000 deliveries. These prospective amendments were added to the trial registry (ISRCTN41244132).

### Randomisation and power calculation

An estimated event rate from the data was used to inform the randomisation sequence and power calculation of the main trial. With so few centres to be randomised there was a risk of unbalance – for example that by chance the centres with the highest event rates might be allocated to the intervention at the end of the study, giving a false impression of a low event rate in centres which received the intervention early, and hence a biased estimate of the treatment effect. Although the planned analysis would correct for this, to further minimize the problem, a restricted form of randomisation was used, so that the rank correlation between the initial event rate, and the order of randomisation was zero. For logistic reasons, the two centres outside Africa: Cap Hatien (Haiti) and Gokak (India) were treated as one centre for randomisation (but not for analysis).

First the overall rate of the primary outcome was determined in the nine clusters and each cluster given a corresponding rank r between 1 and 9. Then a provisional list l of the order of intervention was determined for each cluster using computer-generated random numbers based on an arbitrary random number seed. Spearman’s rank correlation rho between r and l was determined. If the rank correlation was not equal to 0, the list was discarded, and the process repeated until an acceptable order was determined. The first acceptable randomly-generated list was retained. This resulted in a near-zero Pearson’s product-moment correlation of − 0.019 between the event rate and the order of randomisation.

Based on these data, our sample size estimation was carried out by the trial statistician, using Stata version 13.1 and the methods of Hemming and Girling [22]. For the purpose of the power calculation, an assumption that there are at least 4000 deliveries per cluster per month was made (or 8000 per cluster period of 2 months) and at least nine clusters, each observed for 20 months (ten time periods of 2 months each). Our feasibility data indicated a baseline event rate of 1% and we have anticipated a 25% reduction in this to 0.75% post intervention. We would require a total of 2450 outcome events with a coefficient of variation of 0.4 and an Intra Cluster Correlation of 0.0085, to have power of 95%.

## Discussion

This paper aims to describe the mixed methodology feasibility study exploring the acceptability and feasibility of introducing the CRADLE package into routine maternity care in LMIC along with the feasibility of the main trial data collection processes. The key findings are that the intervention can be delivered with high fidelity and dose and incorporated into routine maternity care successfully in different contexts without major adaptation. In addition, we have confirmed that the methods of collecting the main outcome data are feasible and can be maintained for consistency and this data has successfully been used to improve the study design, randomisation and power calculation.

The MRC framework stresses the importance of quality development work to avoid problems with acceptability, compliance, recruitment and retention. Yet methodological research suggests that pilot studies are often poorly performed and few are published [23]. Most published examples of complex interventions demonstrate valuable learning from pilots; for example, the exploratory RCT of an intervention to reduce alcohol related harm demonstrated that whilst the trial was methodologically feasible, poor enthusiasm resulted in low fidelity thus the conclusion that the intervention would need to be enforced in future work [24]. However, published description of its use in the field of maternity and in LMIC is scarce.

One key learning from our feasibility study was the practicalities of a pragmatic process evaluation in a low-resource setting. The MRC supporting guidance provides explanation for each of the key dimensions of implementation that could be measured and guides researchers to select the most important questions to investigate. Whilst this is a strength of the framework as it ensures comprehensive evaluation of implementation, it provides little guidance on how to select suitable outcome measures for each dimension. This is especially challenging when working in a resource-poor environment with additional burden of high workload and heterogeneous routine data collection. In this feasibility study, measuring multiple components compared to baseline was not possible within the short time frame. The proportion of people trained centrally and time taken for training were selected as simple surrogates of fidelity and dose. However, in the qualitative work the majority of HCP reported the VSA was easy to use and it was possible to commence use just on reading the training materials so the extent that these measures may impact the effectiveness of the program is unknown. In the main trial the number of core components delivered in training and how they were adapted to context were added to the measure of fidelity.

The MRC Guidance advises using the logic model to identify causal assumptions and guide selection of research questions. In the CRADLE Logic model the assumed changes to practice include improving access to equipment therefore in the trial we will capture the proportion of HCP that have access to a working BP machine before and after implementation. A further assumed change is improving vital sign measurement and management of complications. In this feasibility study, the action log of referral practice was poorly received and completed due to the extra burden of work it demanded. For the main trial, we chose to explore change in practice during the qualitative work rather than during structured observation due to the large variety of normal practice in different countries. The qualitative work also highlighted that number of women referred to higher level care was a potential key moderator. Therefore, after stakeholder discussion across our sites we agreed to measure the proportion of women presenting for maternity care that had their blood pressure measured and the proportion that were referred to higher level care. Due to the intensity of this data collection it was agreed this would be measured for a period of 4 weeks before implementation and 4 weeks, 3 months after the device was introduced to allow time for familiarization of the VSA in routine maternity care. We found the process of identifying potential factors that may impact on the success of the trial and tailoring outcome measures accordingly was achievable for a team of predominantly newcomers to the field of implementation. It was also beneficial in assuring that assumptions are shared between stakeholders.

### Strengths and limitations of the study

We describe the feasibility testing of the CRADLE intervention prior to a fully powered trial. This was guided by the MRC guidance and was undertaken over a 6-month period; results are therefore applicable to others working in restricted funding periods. Feasibility and pilot research is often skipped or inadequate to fully inform the main trial and rarely published to inform others researching similar areas. In a situation where a complete development phase is not possible due to the stepped wedge design we demonstrated that our event rate in the main trial sites is sufficient to have adequate power. We undertook formal qualitative analysis to explore the acceptability of the program components and feasibility of implementation. We did not collect cost-effectiveness data in the feasibility study but will collect this data for an economic evaluation in the full trial. We did not assess knowledge of vital signs measurement prior to training and therefore results should be interpreted with caution.

## Conclusions

This feasibility study demonstrates that the components of the intervention were acceptable and the methods of implementing successful. Qualitative work identified key moderators that have informed the main trial process evaluation. Changes to the training package, implementation strategy, study design and processes were identified to optimize the main trial implementation. Carrying out these changes in practice, (for example to the data collection forms, database and graphics of the training materials were challenging) within the six-month allocated time frame of this feasibility study. If larger changes had been required, for example to the components of the intervention which would require re-testing, this would not have been possible. Funders recommending that a development phase is incorporated into a standard 3-year funding period might therefore take into consideration the time taken to undertake the work, analyse results and incorporate changes into the study to ensuring meaningful improvements can be made.

Concurrently assessing the feasibility of the trial outcomes in the 10 main trial sites and the implementation processes in geographically distant sites was necessary to avoid contamination of the trial area. Whilst undertaking this in multiple sites required additional work, it allowed for greater understanding of how the intervention could be adapted in each context and how the main trial will run. The MRC Guidance and supporting documents provided a valuable tool to guide the overall design of this study and the development of the process evaluation for the main trial. This study presents a real world worked example that has utilised this guidance to refine the intervention, main trial outcomes and process measures.

## Additional file

Additional file 1: Main Trial Cluster Facilities. Table listing the Primary Investigator and all facilities involved in the main trial in each cluster. (DOCX 21 kb)

## Abbreviations

COREQ: COnsolidated criteria for reporting qualitative research
ERIC: Expert recommendations for implementing change
HCP: Health care provider
LMIC: Low and middle income countrie
MDG: Millennium development goal
MRC: Medical research council
RCT: Randomised controlled trial
SI: Shock index
VSA: Vital sign alert
WHO: World Health Organisation
