# Supplementary material for: Evaluation of a novel vital sign device to reduce maternal mortality and morbidity in low-resource settings: a mixed method feasibility study for the CRADLE-3 trial
Source: BMC Pregnancy Childbirth. 2018 Apr 27;18:115. doi: 10.1186/s12884-018-1737-x (PMC5924508; doi:10.1186/s12884-018-1737-x)
Supplement: Supplementary file 1 — Main Trial Cluster Facilities. Table listing the Primary Investigator and all facilities involved in the main trial in each cluster. (DOCX 21 kb) [file 12884_2018_1737_MOESM1_ESM.docx]

**Main Trial Cluster Facilities**

| **Cluster Primary Investigator and Affiliation** | **Tertiary Facilities** | **Secondary Facilities** | **Primary Facilities** |
| --- | --- | --- | --- |
| Adrian Brown, Maternity Worldwide, UK | St Paul’s Hospital, Addis Ababa, Ethiopia | Ras Desta, Addis Ababa, Ethiopia | Selam Health Center (Woreda 9), Woreda 7 Health Centre (Gulele)/Hidasse, Woreda 10 Health Center (Gulele)/shegole, Free Methodist Health Center, Woreda 5 Health Center, Woreda 2 Health Center Michewe, Addis Ketema Health Center (Addis Ababa), Woreda 5/18 Health Center (Addis Ketema), Woreda 7 Health Center (Addis Ketema), Woreda 7 Health Center (Addis Ketema), Woreda 10 Addis Ketema, Woreda 5 Abebe Bikila, Ras Emiru Health center, Gulele Semen, Simgn Kebede, Kolfe Woreda 2, Addis Gebeya HC, Mikililand Health Centre |
| Francis Gidiri, University of Zimbabwe, Zimbabwe | Mbuya Nehanda Maternity Hospital, Harare, Zimbabwe | Concession District Hospital  Makumbi Hospital | Henderson Clinic, Christon Bank Clinic, Nyabira Clinic, Gwebi College Cllnic, Mount Hampden Clinic, Dzivarasekwa Extension Clinic, St Josephs Clinic, Mabvuku Polyclinic, Warren Park Polyclinic, Hatcliff Polyclinic, Rujeko (DZ) Polyclinic, Parirenyatwa City clinic, Tafara Clinic, Greendale Clinic, Eastlea Clinic, Highlands Clinic, Borrowdale Clinic, Mt Pleasant Clinic, Avondale Clinic, Belvedere Clinic, Mabelreign Clinic, Malborough Clinic |
| Sebastian Chinkoyo, Ndola Teaching Hopital, Ndola, Zambia | Ndola Teaching Hospital | N/A | Chipokota Mayamba Clinic, Chipulukusu Clinic, Commando Camp Clinic, Dola Hill Clinic, Itawa Clinic, Kabushi Clinic, Kalewa Clinic, Kaloko Clinic, Kaniki Clinic, Kawama Clinic, Lubuto Clinic, Main Masala Clinic, Mushili Clinic, Ndeke Clinic, New Masala Clinic, Nkwazi Clinic, Padmodzi Clinic, Peter Singogo Clinic, Prisons Clinic, Railway Surgery, St Dominic Mission Hospital, Tug Argan Clinic, Twapia Clinic |
| Matthew Clarke, Welbodi Partnership, Freetown, Sierra Leone | Princess Christian Maternity Hospital, Freetown, Sierra Leone | Rokupa Government Hospital | Approve School CHC, Haja Neneh, Jenner Wright CHC, Kissy CHC, Konkay CHC, Kuntorloh CHC, Looking Town MCHP, Moyiba CHC, Principal Medical officer Clinic, Ross Road CHC, St. Joseph CHC |
| Carwyn Hill, Hope Health Action, Cap Haiten Haiti | Fort Saint Michel  Hopital, Convention Baptiste d'Haiti,  Justinian University Hospital | Centre de Sante Quartier-Morin | Centre de Sante de Cadush, Centre de Sante de Morne Pele, Centre de Sante Labadie, Centre de Sante Limonade, Centre de Sante Porte Ouverte, Centre de Sante St Charles, Dispansaire St. Louis, Dispensaire de Grand Pre, Unite de Lutte pour la Sante (ULS), Centre de Sante de Madeline |
| Mrutyunjaya Bellad, Jawarharlal Nehru Medical College, KLE University, Belgaum, India |  | Al Shifa Hospital  Arogya hospital Mudalgi  Arogya woman child Hospital,  Mahila and Childrens Hospital  Dhondiba Jadhav Memorial Hospital,  Dr Kattimani Hospital  Ganga surgical and Maternity Clinic  Gokak General Hospital  Gourishankar Hospital Gokak  J G Cooperative Hospital  Jayaratna Hospital,  Kadagalikar Maternity and Children Hospital,  Kappalaguddi Hospital,  KHI Hospital  Masurkar Hospital,  Mudalagi CHC  Muragod Hospital,  Navajeevan Maternity & Nursing Home,  Nayakwadi Hospital,  Shanta Nursing and Maternity Home  Soubhagya Nursing and Maternity Home | Akkatangerhal SC Dasanatti, Akkatangerhal (PHC+ Sub Centres AK Hal I & II, Akkatangerhal SC Iranatti, Akkatangerhal SC Panjanatti, Ankalagi (PHC+ Sub Centres I and II), Ankalagi SC Gujanal, Ankalagi SC Mallapur, Ankalagi SC Suladal, Bairanatti (PHC+ Sub Centres I), Bairanatti SC Sunadholi, Bairanatti SC Tigadi, Balobal SC Hunshyal, Balobal SC Sangankeri, Balobal ( PHC and SC I), Balobal SC Arabhavi, Balobal SC Lolasur, Betageri SC Chikkanandi, Betageri (PHC and SC I), Company Hospital Gokak Falls, Hallur (PHC and SCI and II), Hallur SC Khanatti, Kallolli (PHC and SC I, II), Khanagaon (PHC and SCI), Khanagaon SC DG Hatti, Khanagaon SC Shiltibhavi, Konnur (PHC SC I and II), Konnur SC Godachinamalki, Konnur SC Gokak Falls i and II, Konnur SC Melamatti, Konnur SC Nandagaon, Koujalagi (PHC and SC I and II), Koujalagi SC Kalliguddi, Kulagod (PHC SC I and II), Kulagod SC Dhavaleshwar, Mamadapur (PHC and SCI), Mamadapur SC Maradishivapur, Masaguppi (PHC SCI), Masaguppi SC Dharmatti, Masaguppi SC Vadratti, Melavanki PHC (PHC+ SC-1), Melavanki PHC -SC Maladinni, Melavanki PHC -SC Upparatti, Naganur (PHC SCI), Naganur SC Gurlapur, Naganur SC Mudalagi I and II, Sindhikurabet (PHC SC I and II), Sindhikurabet Ghataprabha I and II, Sindhikurabet SC Dupdhal, Sindhikurabet SC Duradundi, Talakatnal (PHC and SC), Talakatnal SC Gosabal, Talakatnal SC Uddagatti, Tavag (PHC SC I and II), Tavag SC Benachinamardi, Tavag SC Kolavi, Tavag SC Urabinatti, Tukkanatti (PHC SC), Tukkanatti SC PG Mallapur, Tukkanatti SC Rajapur, Yadawad (PHC and SC), Yadawad SC Avaradi, Yadawad SC Girisagar, Yadawad SC Yaragudri |
| Josephat Byamugisha, Makere University, Kampala, Uganda | Mulago and Kawempe Hospital | Lubaga Hospital, Mengo Hospital,  Nsambya Hospital | Kawala Health Centre III, Kisenyi Health Centre, Kisugu Health Centre III, Kiswa Heaslth Centre III, Kitebi Health Centre III, Komamboga Health Centre III, Naguru general hospital, Naguru Teenage Centre, Kibuli Hospital |
| Bellington Vwalika, University of Zambia, Lusaka, Zamba | University Teaching Hospital | Chainama, Chainda, Chawama, Kalingalinga, Kanyama, Mtendere, Chilenje, Chipata, Levy Hospital, Matero Referral, Sikanze | Bauleni, Chaisa, Chazanga, Chelstone, Civic Center, George, Kabwata, Kamwala, Kaunda Square, Matero Main, Ngombe, Prisons, Railway, State House, State Lodge |
| Grace Makonyola, Maternity Worldwide, Malawi | Zomba central Hospital | Balaka , Holy Family, Machinga, Mangochi, St Lukes, Pirimiti | Chingale, Chipini, Magomero, Matawale, Matiya, Mayaka Namikango , Ntaja, Phalombe |
| Julius Wandabwa, Sanyu Africa Research Institute, Mbale, Uganda |  | Mbale regional referral hospital | Ahamadiya. Atuturi hospital, Bubulo, Budadiri, Budaka  Bududa hospital, Bufumbo  Bugobero, Bukedea, Bukiende, Bumadanda, Bumasike, Bunampogo, Bungokho, Busano, Bushikori, Busiu, Busolwe Hospital, Buwangwa, Kadama, Kamonkoli, Kibuku, Kolonyi, Lwangoli, Makhonje, Maluku, Mbale prisons, Mt.elgon hospital, Naiku, Nakaloke, Namakwekwe, Namanyonyi, Namatala, Namawanga, Pallisa hospital, Police 2, Siira HCIII, Sironko, Tirinyi, Wanale |
